# Different symmetries, different mechanisms

**DOI:** 10.3758/s13414-022-02599-9

**Published:** 2022-11-30

**Authors:** Ben J. Jennings, Tzu-Wei Joy Tseng, Marouane Ouhnana, Frederick A. A. Kingdom

**Affiliations:** 1 Centre for Cognitive and Clinical Neuroscience, Division of Psychology, College of Health and life Science, Brunel University London, London, UK; 2 McGill Vision Research, Department of Ophthalmology, Montreal General Hospital, Montreal, Quebec Canada

**Keywords:** Symmetry, Mirror, Radial, Translational, Spatial vision, Visual search

## Abstract

Three common symmetries exist in the natural visual world: (i) mirror symmetry, i.e., reflections around a vertical axis, (ii) radial symmetry, i.e., rotations around a point, and (iii) translational symmetry, i.e., shifted repetitions. Are these processed by a common class of visual mechanism? Using stimuli comprising arrays of Gaussian blobs we examined this question using a visual search protocol in which observers located a single symmetric target patch among varying numbers of random-blob distractor patches. The testing protocol used a blocked present/absent task and both search times and accuracy were recorded. Search times for mirror and radial symmetry increased significantly with the number of distractors, as did translational-symmetry patterns containing few repetitions. However translational-symmetry patterns with four repeating sectors produced search slopes close to zero. Fourier analysis revealed that, as with images of natural scenes, the structural information in both mirror- and radial-symmetric patterns is carried by the phase spectrum. However, for translational patterns with four repeating sectors, the amplitude spectrum appears to capture the structure, consistent with previous analyses of texture regularity. Modeling revealed that while the mirror and radial patterns produced an approximately Gaussian-shaped energy response profile as a function of spatial frequency, the translational pattern profiles contained a distinctive spike, the magnitude of which increased with the number of repeating sectors. We propose distinct mechanisms for the detection of different symmetry types: a mechanism that encodes local positional information to detect mirror- and radial-symmetric patterns and a mechanism that computes energy in narrowband filters for the detection of translational symmetry containing many sectors.

## Introduction

Symmetry is a ubiquitous feature in both natural and artificial images. The most comprehensively studied type of symmetry in vision science is mirror, or ‘reflection’, symmetry (e.g., see reviews by Treder, 2010; Wagemans, 1995, 1997). Formally, mirror symmetry exists if each point within one half of an object is matched by a similar point on the other side of its ‘mirror’ axis, i.e., f(x,y)=f(-x,y).

Less studied but arguably equally important is translational or ‘repetition’ symmetry, as when two or more identical patterns are positioned side-by-side along a given axis (Baylis & Driver, 1994, 2001; Bertamini, 2010; Bruce & Morgan, 1975; Corballis & Roldan, 1974; Julesz, 1971; Kahn & Foster, 1986; Makin et al., 2014; Makin et al., 2012; Tyler & Chang, 1977; Wagemans et al., 1993; Zimmer, 1984). Unlike a pattern constructed from repeated groups of unevenly spaced elements, a special case of translational symmetry is a pattern with evenly spaced elements, the focus of recent studies investigating the perceptual properties of ‘regularity’ (Morgan et al., 2012; Ouhnana et al., 2013; Yamada et al., 2013; Protonotarios et al., 2018; Sun et al., 2019; though see earlier study by Wagemans et al., 1993). Notable among studies of perceptual regularity is the finding that regularity in dot patterns is an adaptable dimension of vision (Ouhnana et al., 2013; Yamada et al., 2013) and that centre and surround patterns with different regularities are subject to perceptual regularity interactions (Sun et al., 2019).

Probably the least studied of the three types of symmetry is radial, ‘rotated’ or ‘centric’ symmetry (Jennings & Kingdom, 2017; Kahn & Foster, 1981, 1986; Palmer & Hemenway, 1978; Royer, 1981; Zimmer, 1984). An object or pattern is said to be radially symmetric if it can be rotated around its origin by some amount (≤ 180°) such that it is indistinguishable from its initial state.

**Fig. 1:**
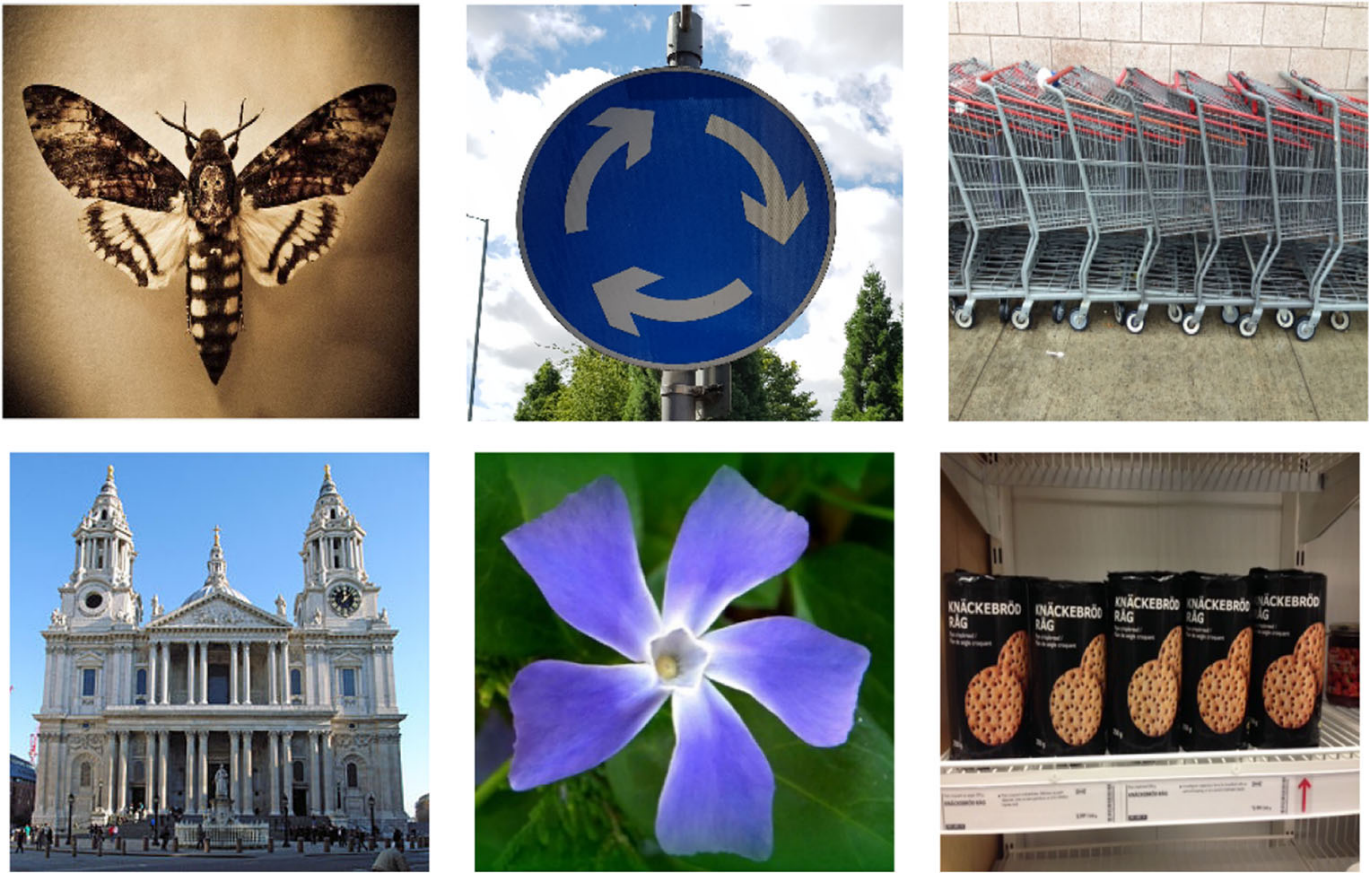
Examples of mirror (**left**), radial (**middle**) and translational (**right**) symmetry. Although the objects themselves are three-dimensional, their two-dimensional projections onto the fronto-parallel plane (and hence also retinal image) are representative of the three types of symmetry studied here psychophysically

Real-world examples of all three symmetry types are shown in Fig. 1.

**Fig. 2:**
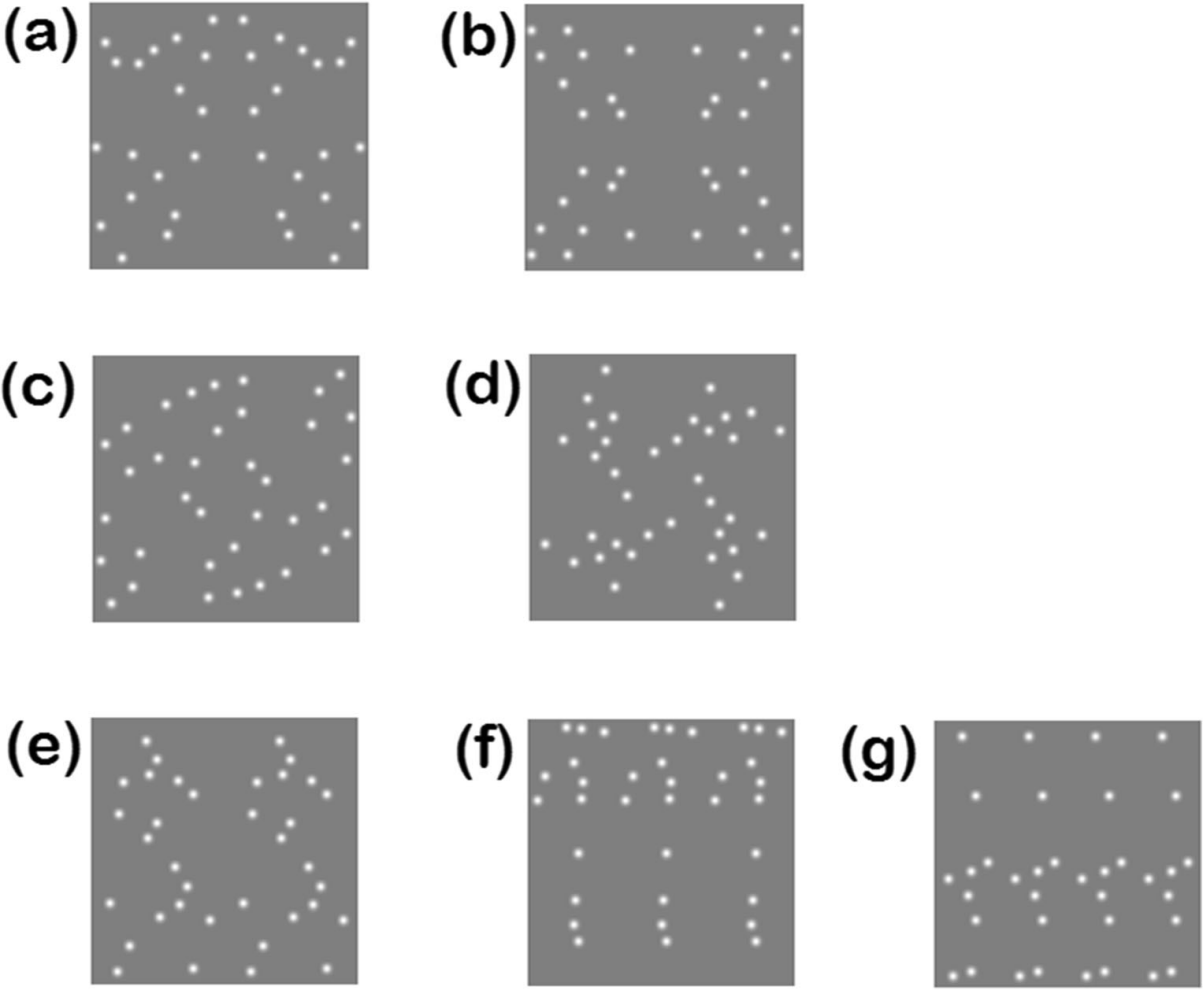
Examples of symmetric target patterns composed of luminance defined Gaussian blobs. Mirror symmetry containing two and four sectors are shown in **a** and **b**, respectively. Radial symmetry containing two and four sectors are shown in **c** and **d**, respectively. Translational symmetry containing two, three and four sectors are shown in **e**, **f** and **g**, respectively

All three types of symmetric structures can be composed of more than two sectors. In the case of mirror symmetry, the pattern may comprise four sectors, with mirror-symmetry exhibited on either side of both the vertical and horizontal axes (see Fig. 2b). In the case of translational symmetry, one can have any number of repeating patterns, as too can radial symmetry, for example a starfish with five identical arms.

A number of studies have attempted to compare sensitivity to two or more of mirror, translational and radial symmetry (Baylis & Driver, 1994, 2001; Bertamini, 2010; Bruce & Morgan, 1975; Corballis & Roldan, 1974; Julesz, 1971; Kahn & Foster, 1981, 1986; Makin et al., 2012; Makin et al., 2014; Palmer & Hemenway, 1978; Royer, 1981; Zimmer, 1984; the pre-1995 studies are reviewed by Wagemans, 1995, 1997). These studies have employed a variety of types of stimuli, for example, patterns made from just a few dots, a large number of dots, line elements and closed shapes; a variety of viewing conditions, for example, foveal versus eccentric; a variety of judgements, for example, symmetry detection versus type of symmetry; and a variety of measures, for example, reaction times, thresholds, electroencephalographic recordings and fMRI. A comprehensive review of the methods, results and conclusions of these studies is beyond the scope of the present communication. However, while the devil lies in the detail, these studies have by-and-large found greater sensitivity to mirror compared to either translational or radial symmetry (e.g., as concluded by Makin et al., 2014; Wagemans, 1995, 1997).

While there are a handful of studies that have employed a visual search paradigm to determine whether target shapes with bilateral symmetry ‘pop-out’ from non-bilaterally symmetric distractors (e.g., Hulleman et al., 2000; Olivers & Van Der Helm, 1998), to our knowledge no studies have used a visual search paradigm to compare sensitivity to mirror, radial and translational symmetry. Given the biological importance of all three types of symmetry for fauna as well as for interactions between fauna and flora, a visual search comparison of the three symmetry types would seem overdue.

In this communication we have employed a visual search paradigm to determine whether all three types of symmetric patterns can be detected pre-attentively. In doing so this has helped to establish the extent to which all three types of symmetric are detected by a common class of mechanism. A brief report of this study has been given elsewhere (Jennings & Kingdom, 2018).

## General methods

### Observers

Twelve observers (11 naïve and one author, TT), aged between 20 and 36 years, took part in the first experiment. A different set of ten naïve observers took part in the second experiment. All observers had normal 6/6 vision (correction was worn if required). Data was collected in accordance with the Declaration of Helsinki (v5) and the Research Ethics Board of the Research Institute of the McGill University Health Centre.

### Equipment

All experiments were conducted on an iMac (Apple Inc) with a Retina 4K display running the Snow Leopard operating system. The stimuli were generated and displayed using custom software developed in MatLab (MathWorks Inc., Matlab, Natick, MA, USA) running PsychToolbox version 3 (Brainard, 1997; Kleiner et al., 2007; Pelli, 1997). The display was driven at 60 Hz with a resolution of 4,096 x 2,304 pixels. During testing observers were positioned 60 cm from the display in a dimly lit room. Observers submitted responses through an external keypad connected via a USB 3.0 port.

### Stimuli

The visual stimuli were composed of circular Gaussian patches (‘blobs’), defined as luminance increments relative to a mid-grey background, with a standard deviation of ~0.057 degrees of visual angle, giving a radius of ~0.14 degrees of visual angle. Blobs were arranged to produce the mirror, radial and translational patterns. Blobs were not allowed to overlap and all stimuli were equal in size.

To create the mirror-symmetric patterns with two sectors, i.e., with one vertical mirror symmetric axis (see Fig. 2a), half the blobs were pseudo-randomly positioned over the left-hand half of the axis, while the other half were positioned in corresponding locations on the right-hand half of the axis. To create the mirror symmetric stimuli with four sectors, a quarter of the blobs were first pseudo-randomly distributed within the top-left-hand quadrant of the stimulus, then their mirror-opposites were positioned in the top-right-hand quadrant, i.e. across a vertical mirror axis. The final half of the blobs were then positioned in the bottom two quadrants as mirror-opposites across a horizontal axis. An example is illustrated in Fig 2b.

Radial symmetric stimuli, with *s =* 2, 3 or 4 sectors, were created by pseudo-randomly positioning the blobs over 1/*s* of the stimulus area then positioning the remaining blobs via rotations of the first sector through 360/*s* deg to fill the remaining *s*-1 sectors. The translational patterns with *s* = 2, 3 or 4 sectors were created by pseudo-randomly distributing 1/*s* of the blobs over 1/*s* width of the stimulus area then copying this sector onto the remaining *s-*1 sectors. For both the radial and translational patterns, as the total number of blobs was 36 they could always be divided equally with no remainder between *s* = 2, 3 or 4 sectors.

### Search task

**Fig. 3:**
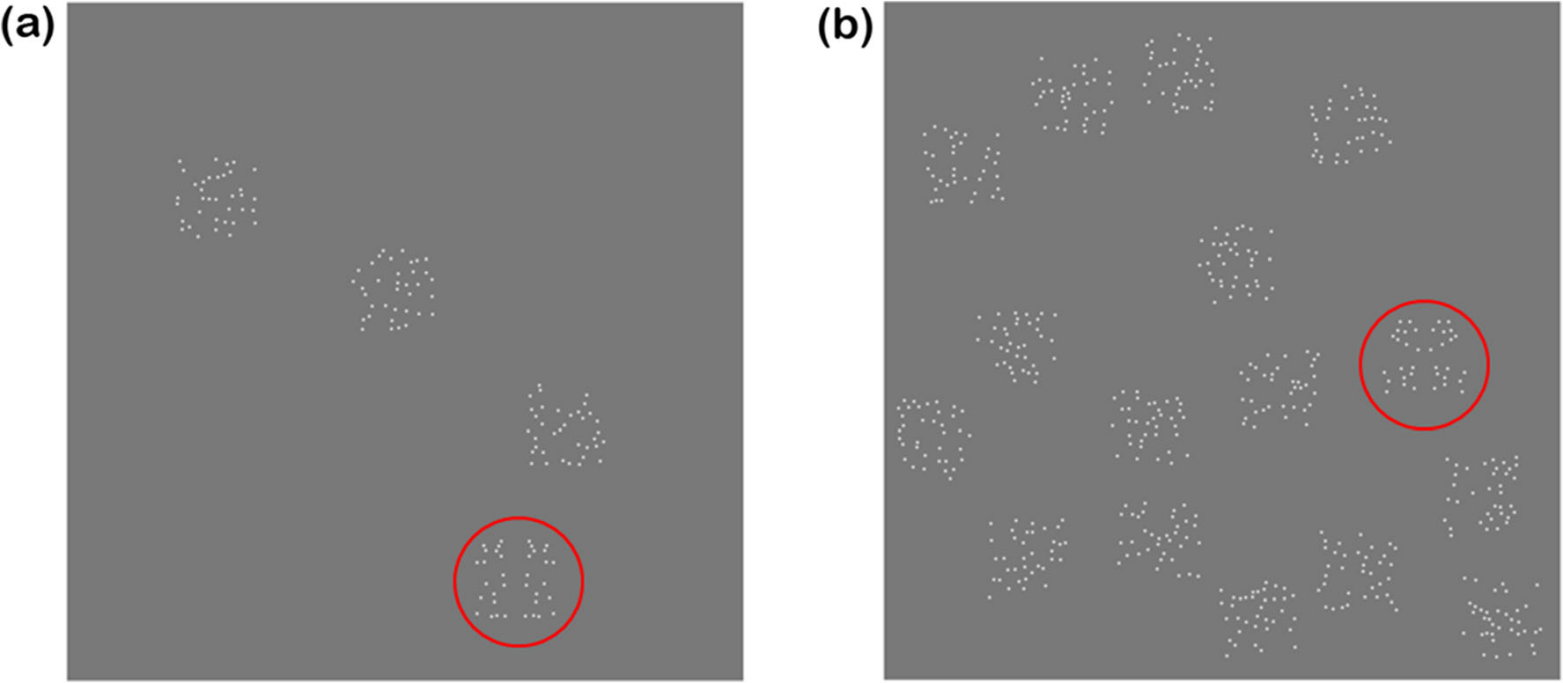
Example search arrays with set sizes of (**a**) 4 and (**b**) 16. Both examples contain a mirror symmetric target highlighted with a red circle (not present during testing)

A traditional visual search paradigm was employed, in which a target was present on 50% and absent on the other 50% of trials (Treisman & Gelade, 1980). The observers’ task was to respond via a button press indicating if the target pattern was present. Different set-sizes of target plus non-target distractor elements were employed within each block, with set sizes of 1, 2, 4, 8 and 16. For example, on a target present trial with a set size of 8 the target plus seven distractors elements would be presented. The distractor elements comprised pseudo-randomly arranged blobs. The target and distractor elements were arranged pseudo-randomly on each trial within the central region of the display, covering 1,400 x 1,400 pixels (targets and distractor patches were 176 x 176 pixels in size). In total each block contained 100 trials, allowing for each set size to be tested 20 times (ten with and ten without the target patch present). Figure 3a and b show example search arrays with set-sizes of 4 and 16, respectively, for two target present trials. The red rings for illustration only indicate the locations of the target patterns amongst the random distractor patches.

For each trial the search time and accuracy were recorded. Feedback in the form of an audible beep was given for an incorrect response. Data were analyzed by extracting median search times for each set-size per condition for both the target-present and target-absent trial types along with the proportion of correct responses.

## Results

**Fig. 4:**
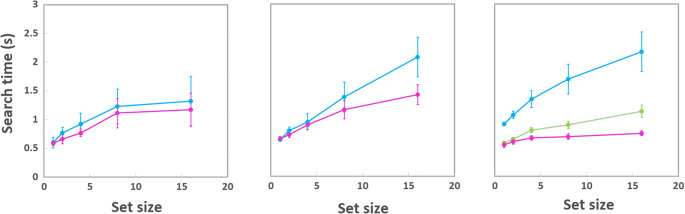
Mirror symmetry with 2 (blue) and 4 (magenta) sectors (left), radial symmetry with 2 (blue) and 4 (magenta) sectors (middle) and translational symmetry with 2 (blue), 3 (green) and 4 (magenta) sectors (right). Error bars are ± 2 SE

The data were analyzed using Welch’s t-tests, for the translational condition a Bonferroni correction (a factor of 3) was applied and corrected p-values are presented for this condition, all reported effect sizes are Cohen’s d values. Figure 4 plots mean search times as a function of set size for all stimuli types (from left to right mirror, radial and translational). For all conditions no significant correlations (all ps > .05) were found between search time and accuracy, indicating no speed-accuracy tradeoffs existed.

A significant correlation existed between search times and set size for all conditions. For mirror symmetry r = 0.92, p = .030 (2 sectors) and r = 0.81. p = .018 (4 sectors). For radial symmetry r = 0.95, p = .013 (2 sectors) and r = 0.91, p = .031 (4 sectors). And for translational symmetry r = 0.97, p < .0056 (2 sectors), r = 0.98, p < .0.003 (3 sectors), and r = 0.96, p < .009 (4 sectors).

The search data were fitted using linear regression (search time vs. set size) and the search slopes (gradients) estimated. The slopes for each condition, fitted to the mean data for each condition are: mirror (2 sectors): 0.045, mirror (4 sectors): 0.040, radial (2 sectors): 0.094, radial (4 sectors): 0.051, translational (2 sectors): 0.97, translational (3 sectors): 0.35 and translational (4 sectors): 0.012 (all in s/item). No difference in slopes between the two 2- and 4-sector mirror-symmetry conditions was found (t(11) = -0.0049, p = .50, d = 0.001). Between the two radial conditions a significant difference in slope existed (t(11) = 6.38, p < .0001, d = 1.8). For the translational condition between the 2- and 3-sector condition no difference was found between slopes (t(11) = 1.97, p_corrected_ = .29, d = 0.40); between the 2- and 4-sector condition the difference failed to be significant after correction, however a medium effect size was found ((t(11) = 1.97, p_corrected_ = .10, d = 0.57); finally a significant difference existed between the 3- and 4-sector conditions ((t(11) = 2.79, p_corrected_ = .026, d = 0.81).

### Phase, amplitude and a filter-rectify-filter model

**Fig. 5:**
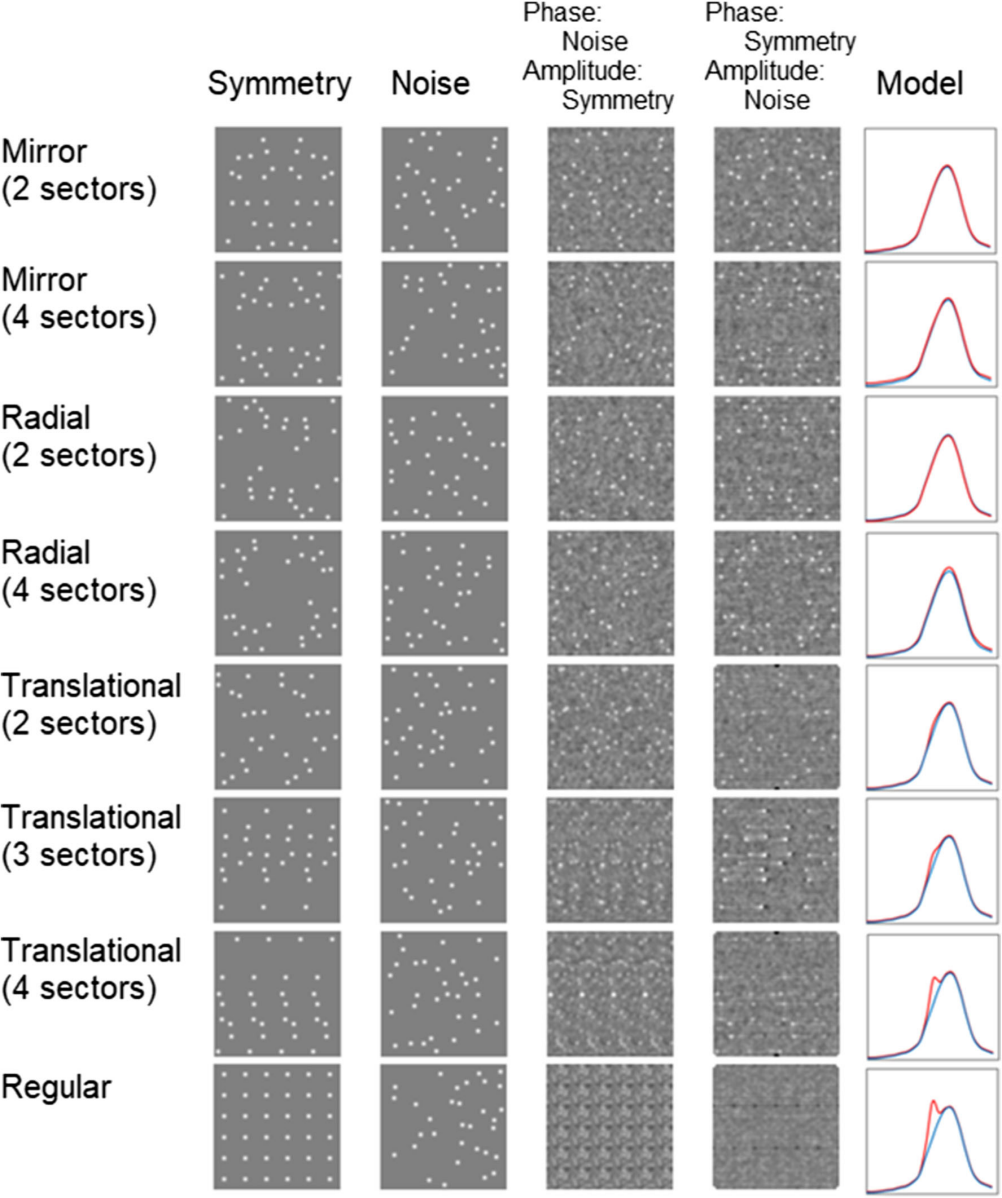
Each row shows the following for a different symmetric pattern: Column 1: the symmetric pattern under consideration. Column 2: a noise pattern. Column 3: the result of combining the phase information from the noise with the amplitude information from the symmetric pattern. Column 4: the result of combining the amplitude information from the noise with the phase information from the symmetric pattern. Column 5: the output of the filter-rectify-filter (FRF) model, the blue plots the model output for the noise patterns, while the red plots the output for the symmetric patterns

The spatial structure contained in an image of a natural scene is carried primarily by the Fourier phase not amplitude spectrum (Oppenheim & Lim, 1981; Piotrowski & Campbell, 1982). Based on a similar analysis by Ouhnana et al. (2013, Fig. 9) for patterns differing in regularity, Fig. 5 shows the result of swapping the phase and amplitude spectra of symmetric and random patterns. It can be seen that the symmetric structure is predominantly carried by the phase spectra for the mirror and radial patterns, as well as for the translational patterns with two sectors. However, as the number of translational sections increases from two, a switch occurs and the pattern becomes more discernable in the amplitude spectra (column 3). The bottom row of Fig. 5 shows the result of the analysis conducted by Ouhnana et al. (2013), showing that the structure of a perfectly regular pattern is carried by the amplitude spectrum. This analysis is suggestive of the idea that patterns composed of many repetitions along a given axis are detected by a mechanism that relies on information from the Fourier amplitude spectrum, whereas small-repetition-number translational, as well as mirror and radial patterns with any number of sectors rely on information from the phase information.

The fifth column of Fig. 5 plots the output of a simulation based on a filter-rectify-filter (FRF) model (Chubb & Sperling, 1988; Chubb & Landy, 1991; Graham et al., 1992; Graham, 2011; Wilson et al., 1992). The standard FRF model is based on the following stages. First the input image is convolved with a series of ‘first-stage’ Gabor filters (that simulate simple cells) with different orientation and spatial frequency selectivities. An intermediate stage then full-wave rectifies (or squares) the outputs of the first-stage filters to make all their responses positive, thereby preventing the outputs from cancelling when pooled within each of the receptive field sub-regions of the ‘second-stage’ filters. These second^-^stage filters, which are larger than their first-stage counterparts, thus serve to pool the energy responses from the first stage.

The graphs on the right of Fig. 5 plot the second-stage responses as a function of the (log) spatial frequency of the first^-^stage filters. Two curves are plotted for each stimulus type, blue for the noise and red for the symmetric patterns. There are no differences between the symmetric and noise pattern curves until a secondary peak in the red curve starts to emerge with the 3-sector translational pattern, increasing with 4 sectors and being most pronounced for the completely regular pattern, the last of these confirming a similar analysis conducted by Ouhnana et al. (2013) and Protonotarios et al. (2018).

### Searching for a regular pattern

Based on the output of the FRF model, a prediction can be made regarding the performance in a search task using a regular pattern as target. If the secondary peak in the energy function (Fig. 5) is being utilized by the visual system to identify the translationally symmetric target, then a completely regular pattern should be even more salient than the translational patterns and hence produce an even lower measured search slope.

**Fig. 6:**
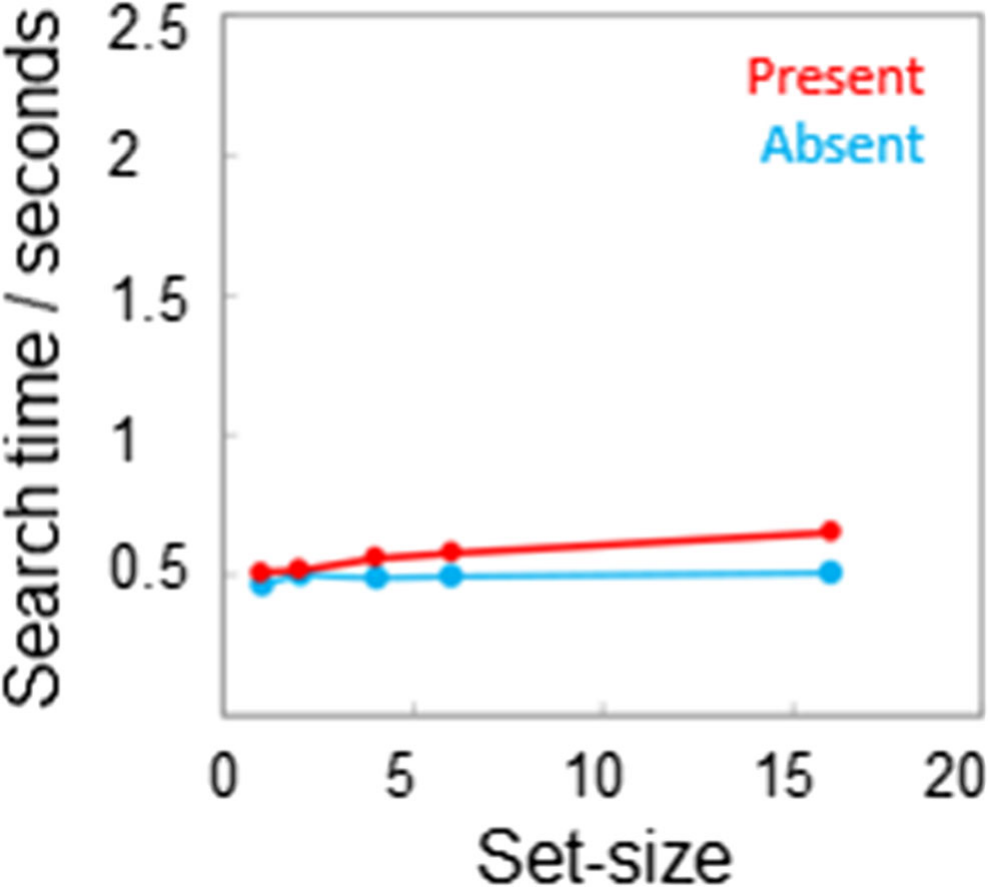
Search times as a function of set-size for the regular patterns, the target present and absent conditions are plotted in red and blue, respectively. SEs have been omitted as they are approximately the size of the data points

This prediction was tested with a new group of observers (n = 10), using an identical testing procedure as that described in the section on the search task. The data are presented in Fig. 6; again, search times for correct responses are plotted as a function of set-size, with red for target present and blue for target absent trials. Search slopes are effectively zero. Accuracy for both conditions (target present and absent) was also found to be independent of set-size, with an average proportion correct of 0.98.

## Discussion

The main findings of the current study are:

(i) No difference was found in search times between 2- and 4-sector mirror-symmetric patterns.
(ii) Search times were shorter for 4-sector compared to 2-sector radially symmetric patterns.
(iii) Search times for 2-, 3- and 4-sector translationally symmetric patterns decreased with the number of sectors.
(iv) Swapping the Fourier phase and amplitude spectra of symmetric and random patterns reveals that for mirror, radial and translational patterns up to two repeating sectors symmetry information is carried by the phase.
(v) Swapping the phase and amplitude spectra for random and translational symmetry patterns with more than two repeating sectors, or for random and regular patterns, indicates the symmetry information in these cases is carried by the amplitude spectra.
(vi) Search times for regular patterns are efficient, i.e., independent of set-size.

These results suggest that there are two types of mechanism involved in detecting dot symmetry patterns in visual search tasks. One mechanism utilises phase information, i.e. explicitly encodes dot spatial relationships, and requires attention, i.e. does not result in pop-out. This mechanism is used to detect mirror- and radial-symmetric patterns, as well as translational patterns containing few repeating sectors. On the other hand, another, pre-attentive mechanism utilises amplitude information, i.e., the pattern of energy across spatial frequency and/or orientation. This mechanism is responsible for the detection of translational symmetry and explains why translational symmetries containing many repeating sectors as well as complete regular patterns are pop-out features. Although the regular translational symmetry pattern in Fig. 5 also possesses four axes of mirror-symmetry (horizontal, vertical, -45°, +45°) and 4 sectors of rotational-symmetry, the psychophysical results with mirror/radial-symmetry patterns suggest that these are not the features mediating its detection, which is efficient, at least in our displays. Of course, our analysis leaves open the question of how exactly the dot spatial relationships are encoded by the phase-sensitive symmetry mechanism, and in answer to this question numerous theories, too many to be described in detail here, have been advanced (reviewed by Treder, 2010; Wagemans, 1995, 1997). Moreover, precisely how the amplitude spectra of regular and multi-sector translational patterns are used for their detection has yet to be determined, though a number of ideas have recently been advanced (Sun et al., 2019).

Previous studies have identified a set of stimulus dimensions that facilitate efficient search of targets among distractors (e.g., color, orientation, size, etc.), as evidenced by an absence of an effect of the number of distractors on search times. These dimensions have been referred to as guiding attributes (Wolfe & Horowitz, 2004) or pre-attentive features (Treisman & Gelade, 1980). Based on the data presented here, demonstrating that high-sector number translational symmetry and regular patterns support efficient search, we propose that spatially regular structures, i.e., regular grids, should be added to this list. On the other hand, our data suggest that mirror- and radial-symmetric patterns are not detected pre-attentively. Finally, our Fourier phase-amplitude swap analysis is in keeping with the idea that for textured stimuli, it is those patterns that produce different energy responses in filters tuned to different luminance spatial-frequencies and/or orientations that can be effortlessly discriminated (Chubb & Landy, 1991; Graham, 2011).

## Declarations

### Open Practices Statement

This study was not pre-registered; however, data are available on request from the corresponding author.
